# Mutuelles de santé à Bukavu en République Démocratique du Congo: facteurs favorables à l’utilisation des services de santé par des adhérents

**DOI:** 10.11604/pamj.2020.35.100.20441

**Published:** 2020-04-07

**Authors:** Justine Bashi, Drissa Sia, Eric Tchouaket, Safari Joseph Balegamire, Hermès Karemere

**Affiliations:** 1 Bureau Diocésain des Œuvres Médicales de Bukavu, Bukavu, République Démocratique du Congo; 2 Département des Sciences Infirmières, Université du Québec en Outaouais, Saint-Jerôme, Canada; 3 Ecole de Santé Publique, Université de Montréal, Montréal, Quebec, Canada; 4 Faculté de Médecine, Ecole Régionale de Santé Publique, Université Catholique de Bukavu, Bukavu, République Démocratique du Congo

**Keywords:** Mutuelle de santé, utilisation des services, déterminants, Bukavu, Sud-Kivu, République Démocratique du Congo, Mutual health insurance, use of services, determinants, Bukavu, South Kivu, Democratic Republic of the Congo

## Abstract

**Introduction:**

La présente étude s'intéresse aux déterminants de l'utilisation des services de santé par des adhérents aux trois mutuelles de santé dans la ville de Bukavu en République Démocratique du Congo.

**Méthodes:**

L'étude, de type descriptif transversal, est une enquête de perception menée auprès des utilisateurs des services de santé affiliés aux mutuelles de santé dans les zones de santé de Bukavu. L'encodage et l'analyse statistique ont été réalisés avec le logiciel Epi Info version 2010.

**Résultats:**

Les principaux déterminants de l'utilisation des services de santé par des adhérents aux mutuelles de santé sont: le lieu de résidence de l'adhérent, le niveau d'instruction du responsable du ménage, l'expérience antérieure des soins dans la structure sanitaire partenaire de la mutuelle de santé, la réputation de la structure partenaire des mutuelles de santé et la capacité des ménages à payer le ticket modérateur.

**Conclusion:**

La présente étude révèle, qu'au-delà de la barrière financière, le développement d'une mutuelle de santé devrait permettre de promouvoir une meilleure régulation du ticket modérateur et une bonne qualité des soins pour satisfaire les besoins en soins de ses adhérents. Les facteurs qui ressortent de l'étude en tant que principaux déterminants de l'utilisation des services de santé des adhérents à une mutuelle de santé ne sont pas souvent pris en compte lors de l'implantation des mutuelles de santé dans des contextes similaires à ceux de Bukavu.

## Abstract (en)

**Introduction:**

This study highlights the determinants of the use of health services by adherents to the three mutual health insurances in the town of Bukavu in the Democratic Republic of the Congo.

**Methods:**

We conducted a descriptive cross-sectional study, based on a perception survey among users of healthcare services affiliated to the mutual health insurances in the Bukavu health zones. The encoding and statistical analysis were carried out using the Epi INFO version 2010 software.

**Results:**

The main determinants of the use of healthcare services by adherents to the mutual health insurances are: the member’s place of residence, the level of education of the head of household, the previous experience of care in the healthcare structure partner of the mutual health insurances, the reputation of the structure partner of the mutual health insurances and the ability of households to pay the user fee.

**Conclusion:**

This study highlights that, beyond the financial barrier, the implementation of a mutual health organisation should promote a better regulation of the user fee and a good quality of care to meet the care needs of members. The factors emerging from the study as a major determinant of the use of health services by adherents to a mutual health insurance are often not taken into account in the implementation of mutual health insurance in contexts similar to those of Bukavu.

## Introduction

En République Démocratique du Congo (RDC), la prise en charge financière des soins de santé est majoritairement assurée par des individus, en lieu et place des systèmes de financement collectif comme cela est le cas dans les pays dits développés [1]. Cette situation limite l'accessibilité aux soins pour environ 70% des congolais vivant dans un contexte d'extrême pauvreté [2] et ainsi exposés aux graves problèmes de santé. La prévention et le dépistage sont des pratiques beaucoup moins répandues parmi les personnes les plus pauvres [3, 4]. Trop souvent, ces personnes se rendent aux services de santé au stade de complications de leur maladie, quand ils n'ont plus d'autre alternative. Plusieurs initiatives ont été mises en place en RDC pour faire face à la faible accessibilité aux soins par la population parmi lesquelles le financement communautaire des soins, la mutualisation des risques d'appauvrissement en cas de maladie au travers d'un prépaiement des soins [5-8] et le financement basé sur la performance [9-12]. Certaines de ces initiatives ont échoué [6] et d'autres se sont poursuivies sans atteindre leurs objectifs à cause de l'évolution contextuelle influencée par des facteurs d'organisation managériale, économiques, sociaux et politiques difficiles [13]. La présente étude s'intéresse à trois mutuelles de santé de Bukavu, une des villes de la RDC. Une mutuelle de santé (MUSA) pourrait être définie comme étant une association de personnes physiques, à but non lucratif, dont la base de fonctionnement est l'entraide et la solidarité, et qui, au moyen des cotisations des membres, et à partir de leurs décisions, mène en leur faveur et en celle de leurs familles des actions de prévoyance des risques liés à leurs problèmes de santé [14].

Une étude sur l'implantation des MUSA au Rwanda a démontré que les ménages de grande taille (plus de 5 personnes) et ceux ayant un revenu relativement plus élevé y adhèrent plus. Dans la même étude, les membres de la mutuelle utilisent plus les services de santé que les non membres, dépensent moins pour leurs soins de santé et se fidélisent à la mutuelle au fil des années [15]. Le Rwanda a entre temps évolué vers une assurance maladie obligatoire qui devra trouver sa place dans les systèmes de financement adaptés aux caractéristiques des pays à faible revenu [16]. Bien que le mouvement mutualiste n'ait cessé de se développer en Afrique, le pourcentage de la population couverte n'atteint généralement guère plus d'1%. Les faibles taux de pénétration des MUSA remettent parfois en question leur viabilité [17] et sont essentiellement associés à la qualité des soins de santé, à la confiance des habitants dans la réussite d'une MUSA et à la capacité financière des communautés [1]. Plutôt que d'analyser les déterminants de l'adhésion à une MUSA, largement étudiés par Defourny *et al.* [17], nous avons pris la perspective d'étudier les déterminants de l'utilisation des services de santé par des adhérents à une MUSA. Notre question est ainsi de savoir les facteurs favorables d'utilisation des services de santé par les adhérents à la MUSA dans la ville de Bukavu.

## Méthodes

### Description du terrain d'étude

L'étude a eu lieu à Bukavu, une ville située dans la province du Sud-Kivu à l'Est de la République Démocratique du Congo et dont la population avoisine 876 917 habitants en 2015. Elle comprend trois communes: Bagira-Kasha (127 160 habitants), Ibanda (412 996 habitants) et Kadutu (336 761 habitants) [18].

### Description des mutuelles de santé étudiées et de leur evolution

Les MUSA au Sud-Kivu sont nées en 1990 sur l'initiative de l'église catholique de Bukavu au travers de son bureau diocésain des œuvres médicales (BDOM) pour améliorer l'accès aux soins. Une cellule d'appui aux mutuelles de santé (CAMS) mise en place au sein du BDOM consolide et accompagne techniquement le processus d'implantation des mutuelles de santé dans les zones de santé. Les MUSA visent essentiellement à affilier l'ensemble des membres des ménages de leur rayon d'action et à garantir à leurs adhérents l'accès facile aux soins de santé de qualité seulement dans les structures de santé implantées dans leur rayon d'action. La cotisation annuelle par membre est de 3,5 dollars américains lorsqu'il s'agit d'un enfant de 5 ans et moins et elle est 7 dollars américains pour chaque membre âgé de plus de 5 ans. Lors des soins, la MUSA rembourse 80% des frais d'hospitalisation et 50% des frais si les soins sont administrés en ambulatoire. La MUSA ne prend en charge que 4 épisodes-maladie par adhérent et par an. La tarification des soins est généralement forfaitaire dans les structures des soins conventionnées par la MUSA. Les forfaits sont les mêmes pour les trois zones de santé. Les frais complémentaires aux 80% et aux 50% doivent être payés par le patient et constituent le ticket modérateur. Le circuit des soins doit être respecté par les patients. Certaines dépenses en soins ne sont pas remboursées par les MUSA. C'est notamment les frais concernant les soins en cas de maladie chronique (diabète, hypertension artérielle, cancers, tuberculose, etc.), la chambre privée d'hospitalisation, les lunettes, les prothèses, la maternité d'une fille dépendante d'un ménage, le transfert à l'étranger pour des soins, l'interruption volontaire de la grossesse, les soins d'un membre n'ayant pas fini de payer la totalité de la cotisation et les médicaments spécialisés. Les MUSA interviennent au niveau des formations sanitaires partenaires pour influencer la qualité des soins grâce à leurs conseillers médicaux, sur la base des conventions préalablement signées. Ces conventions déterminent le paquet de soins assurés, le prix facturé des soins, les modalités de paiements des frais et les mécanismes de vérification des soins administrés. Chaque MUSA a ses structures des soins conventionnées, différentes d'une zone de santé (ZS) à l'autre et limitant ainsi la portabilité de l'assurance aux structures uniquement conventionnées par la MUSA.

### Type d'étude, population à l'étude et variables utilisées

L'étude menée est de type descriptif transversal. C'est une enquête de perception auprès des utilisateurs des services de santé affiliés à la MUSA. L'étude a été réalisée dans les zones de santé (ZS) d'Ibanda, Bagira-Kasha et Kadutu de mars 2015 à octobre 2015. Chaque ZS possède une MUSA soit au total trois MUSA concernées par l'étude. La population de l'étude était constituée des adhérents à chacune des trois MUSA, desservies par plusieurs formations sanitaires (FOSA) qui sont les centres de santé, les centres hospitaliers et les hôpitaux généraux de référence. Au total, 87 structures des soins étaient partenaires aux MUSA dont 25 dans la ZS d'Ibanda, 42 dans la ZS de Kadutu et 20 dans la ZS de Bagira-Kasha. Le choix des adhérents aux MUSA était effectué par la technique accidentelle. Seules ont été incluses les personnes présentes dans les formations sanitaires partenaires des MUSA les jours de l'enquête et répondant aux critères ci-après: être membre d'une mutuelle de santé, être responsable d'un ménage (mère ou père) et avoir donné son consentement éclairé et volontaire. La taille minimale de l'échantillon était déterminée grâce à la formule de Schwartz [19], ensuite majorée de 10% afin de minimiser le risque d'erreur associé aux non répondants potentiels. Au total, 422 sujets avaient pris part à l'étude. La variable dépendante est l'utilisation des services de santé au cours des 4 derniers épisodes-maladies concernant des adhérents. Les variables indépendantes sont les suivantes: les caractéristiques sociodémographiques des répondants; la prise en charge de toutes les pathologies par la MUSA; la composition des ménages; la satisfaction des adhérents par rapport à la qualité des soins; la réputation des structures sanitaires partenaires à la MUSA et la capacité à payer le ticket modérateur. Les caractéristiques sociodémographiques concernent essentiellement l'âge, le sexe, l'état civil, la religion, le niveau d'étude, la taille du ménage et la profession. La religion a été catégorisée en deux pour des raisons d'analyse (religion chrétienne et autre religion).

### Collecte des données

La collecte des données avait recouru aux entrevues individuelles avec les responsables des ménages sélectionnés et à la revue documentaire. A l'aide d'un questionnaire semi-structuré, des informations avaient été collectées concernant les caractéristiques sociodémographiques des répondants; les raisons de non adhésion aux MUSA par certains membres du ménage; le niveau d'adhésion des membres par ménage; le niveau d'utilisation de services de santé par chaque membre de la famille adhérent par an; les problèmes sanitaires non pris en charge par les MUSA; le degré de satisfaction des répondants et les propositions visant l'amélioration de l'utilisation des services par les adhérents. La revue documentaire avait consisté à collecter des informations concernant le fonctionnement des MUSA et les statistiques des adhérents aux MUSA à partir des rapports des centres de santé et des MUSA étudiés.

### Encodage et analyse des données

L'encodage et l'analyse statistique avaient été réalisés avec le logiciel Epi INFO version 2010 et le seuil de signification statistique fixé était de 5%. Les proportions ont été utilisées pour décrire les variables en catégories; la moyenne et la déviation standard (DS), lorsque la variable était quantitative continue. Le test chi carré de Pearson a été utilisé dans l'analyse des tables de contingence. Lorsque les attendus étaient supérieurs à 5 dans plus de 20% de cellules, le test de Fischer était utilisé. Les Odds Ratio (OR) et leurs intervalles de confiance à 95% (IC 95%) ont été calculés pour estimer l'association entre les variables d'exposition et les évènements étudiés.

### Considérations éthiques

Le protocole d'étude a été validé par le Comité d'Ethique de l'Université Officielle de Bukavu et a obtenu l'approbation des équipes cadres des zones de santé d'Ibanda, de Kadutu et de Bagira-Kasha. Le consentement volontaire et éclairé de participation à l'enquête a été systématiquement sollicité auprès des sujets de l'étude.

## Résultats

### Évolution des adhésions aux mutuelles de santé à Bukavu

**Figure 1:**
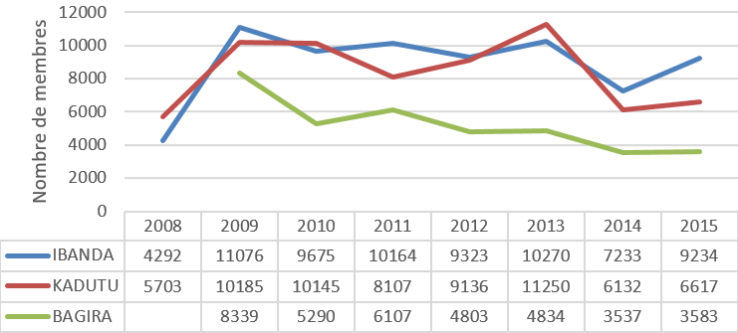
Évolution des adhésions aux mutuelles de santé à Bukavu entre 2008 et 2015

La tendance générale des adhésions est à la baisse entre 2008 et 2014. Rapportée à la population générale, les adhérents à la mutuelle de santé constituent 2% de la population de Bukavu en 2015 (Figure 1).

### Caractéristiques sociodémographiques des personnes interrogées

Les caractéristiques sociodémographiques des personnes interrogées soit 422 personnes sont présentées dans le Tableau 1. On observe que 53% des personnes interrogées étaient plus jeunes (moins de 30 ans); 30% des responsables d'enfants étaient des hommes; la grande majorité des personnes étaient mariées (77%), catholiques (70%) et avec moins de sept personnes dans leur ménage (84%). Le niveau secondaire dans la scolarisation des personnes interrogées est le plus dominant (58%) et 36% des personnes sont au chômage (Tableau 1).

**Tableau 1:** Caractéristiques sociodémographiques des enquêtés

Paramètres	Effectif (%)	M±DS
**Age (ans)**	n= 422 (100,0)	31±8,7
Moins de 30 ans	224 (53,1)	
30 ans et plus	198 (46,9)	
**Sexe**	n=422 (100,0)	
Féminin	294 (69,7)	
Masculin	128 (30,3)	
**Etat civil**	n= 422 (100,0)	
Célibataire	62 (14,7)	
Divorcé	24 (5,7)	
Marié	326 (77,3)	
Veuve	10 (2,4)	
**Religion**	n=422 (100,0)	
Chrétiennes	388 (91,9)	
Autres religions	34 (8,1)	
**Niveau d’étude**	n=422 (100,0)	
Sans niveau	48 (11,4)	
Primaire	57 (13,5)	
Secondaire	244 (57,8)	
Universitaire	73 (17,3)	
**Taille du ménage**	n= 422 (100,0)	
Moins de 7	356 (84,4)	6±2 persons
7 et plus	66 (15,6)	
Profession	n= 422 (100,0)	
Commerçant	92 (21,8)	
**Enseignant**	43 (10,2)	
Salarié de l’Etat	74 (17,5)	
Salarié privé	61 (14,5)	
Sans profession	152 (36)	

### Niveau d'adhésion des ménages et raisons de non adhésion à la MUSA

Dans 193 ménages sur 422 (soit 46%), seulement quelques membres par ménage ont adhéré à la MUSA alors que dans 229 ménages (soit 54%) tous les membres du ménage ont adhéré à la MUSA. Le fait de ne pas tomber malade pour certains membres au sein d'un ménage est la cause de non-adhésion à la MUSA la plus citée (72% des responsables des ménages interrogés). Le Tableau 2 présente les causes de non adhésion pour les 193 ménages concernés (Tableau 2).

**Tableau 2:** Raisons de non adhésion de certains membres d’un ménage à la MUSA

Raisons de non adhésion de certains membres d’un ménage à la MUSA selon les responsables des ménages	Effectif (%) n=193 (100)
Mauvais accueil humain des patients par le personnel soignant	6 (3,1)
Non octroie de crédit par la MUSA	13 (6,7)
Enrichissement des responsables des MUSA (au travers des frais de cotisation) ou des infirmiers des centres de santé grâce (au travers des frais des tickets modérateurs)	17 (8,8)
Certains adhérents ne tombent pas malades	139 (72,0)
Non prise en charge de tous les problèmes de santé	18 (9,3)

### Utilisation des services de santé par des adhérents

La comparaison de l'utilisation des services par un ménage où l'ensemble des membres a adhéré à la MUSA et celle par un ménage dans lequel seulement certains membres ont adhéré ne montre pas de différence statistiquement significative comme illustré dans le Tableau 3. Les raisons de non utilisation des services des soins par les adhérents évoquées par les personnes interrogées (n=422) sont selon l'ordre d'importance de l'automédication (50,4%), la non prise en charge de certains problèmes par les services des soins dont les soins intensifs, l'oxygénothérapie en néonatologie ou certains médicaments (21,8%), la longue distance entre le domicile et la structure des soins (14,5%) et le recours à la prière au travers des chambres ouvertes par des pasteurs, notamment évangélistes (13,3%).

**Tableau 3:** Utilisation des services de santé comparée au niveau d’adhésion à la MUSA

	Utilisation des services nombre (%)	OR non ajusté (IC 95%)	p-value
**Niveau d’adhésion**			
Adhésion de certains membres du ménage	152 (29,6)	0,42 (0,16-1,13)	0,05
Adhésion de tous les membres du ménage	22 (50,0)	1	

### Facteurs influençant les adhérents à utiliser les services des soins

#### Influence des facteurs sociodémographiques

Certains facteurs ont été identifiés comme étant à la base de l'utilisation des services. Ce sont essentiellement le lieu de résidence de l'adhérent et le niveau d'instruction du responsable du ménage pour lesquels les Odds Ratio (OR) et leurs intervalles de confiance à 95% (IC 95%) ont été statistiquement significatifs. Par contre, d'autres facteurs ont été identifiés comme n'influençant pas l'utilisation de service de santé. Ce sont le sexe, la religion, l'état civil, la profession et la taille de ménage (Tableau 4). Sur 422 personnes interrogées, 268 ont utilisé les services au cours de leurs 4 derniers épisodes de maladie. Parmi eux, 85% habitent à Bagira-Kasha, 37% habitent à Ibanda et 62% habitent à Kadutu. Les personnes du niveau d'études secondaire ont utilisé environ deux fois plus les services que ceux du niveau primaire.

**Tableau 4:** Facteurs sociodémographiques ayant influencé l’utilisation des services de santé par les répondants

Variables	Nombre d’individus	(%) ont utilisé	OR non ajusté (IC 95%)	P
**Résidence**	**n=268**			< 0.001^*^
Bagira	40	85,0	3,48 (1,36-8,92)	
Ibanda	102	37,2	0,36 (0,2-0,62)	
Kadutu	126	61,9	1	
**Sexe**				
Masculin	86	54,7	0,92 (0,55-1,54)	0,76
Féminin	182	56,6	1	
**État civil**				0,54
Non en unions	59	52,5	0,83 (0,46-1,49)	
En union	209	56,9	1	
**Religion**				0.55
Non chrétiens	45	60,0	0,82 (0,42-1,57)	
Chrétiens	223	55,1	1	
**Niveau d’étude**				0.03
≤ Primaire	150	19,3	0,54 (0,3-0,95)	
≥ Secondaire	118	30,5	1	
**Profession**				0,74
Sans emploi	88	54,5	0,91 (0,54-1,53)	
Avec emploi	180	56,6	1	
**Taille ménage**				0.36
> 7	31	48,3	0,70 (0,33-1,49)	
≤ 7	237	56,9	1	

#### Influence d'autres facteurs

Satisfaction des adhérents aux MUSA par rapport à la qualité des prestations offertes par les services des soins: la majorité (85,9%) des personnes interrogées ne sont pas revenus aux soins à cause de leur insatisfaction à la suite d’une expérience antérieure (Tableau 5).

**Tableau 5:** L’utilisation des services par rapport à la satisfaction des quatre épisodes qui sont prises en charge par la MUSA

Utilisation des services après une première expérience	Patients ayant retourné		Patients n’ayant pas retourné		Khi^2^	p
	n	%	n	%		
Moins satisfait	20	14,1	121	85,9	2	NS
Satisfait	224	91,1	25	8,9		

Réputation de la structure des soins partenaire de la MUSA: l'étude démontre que 91% des personnes interrogées se déclarent insatisfaites des structures des soins partenaires à leur MUSA (Tableau 6). L'utilisation des services de santé par les ménages membres de la MUSA varie en fonction du choix de la structure partenaire opéré par la MUSA pour ses membres: OR = 81,1(34,9-188,56) avec p < 0,001 (Tableau 6).

**Tableau 6:** Satisfaction des répondants face aux structures des soins partenaires à la MUSA

	N’ont pas utilisé		
	N total (%)	OR (IC 95%)	p-value
Satisfaction par rapport au choix de structure partenaire			< 0,001
Ne sait pas	21 (19,0)	1,92 (0,57-6,49)	
Non	109 (90,8)	81,1 (34,9-188,56)	
Oui	138 (10,8)	1	

Paiement du ticket modérateur: du fait du paiement préalable des frais couvrant le ticket modérateur, 51% des personnes interrogées (n=212) déclarent ne pas accéder aux services de santé. Les ménages qui présentent les difficultés de payer le ticket modérateur avaient 4 fois plus de chance de ne pas utiliser les services de santé (OR=4,1(2,2-9,9), p<0,001).

## Discussion

**Figure 2:**
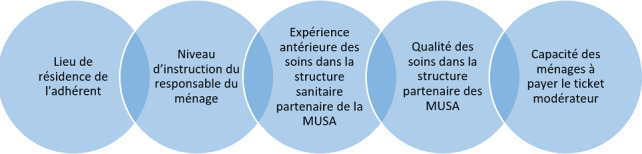
Facteurs favorables à l’utilisation des services des soins dans la ville de Bukavu

La présente étude a pour objectif de déterminer les facteurs favorables à l'utilisation des services par des adhérents à une mutuelle de santé dans la ville de Bukavu. Les limites de cette étude sont de deux ordres. Premièrement, l'absence de collecte d'information auprès du personnel soignant sur le niveau d'utilisation des services de santé par les adhérents aux MUSA et ensuite l'analyse objective de la qualité effective des soins dans les structures sanitaires partenaires aux MUSA. Malgré ces limites, les résultats de cette étude restent valides dont les principaux sont présentés dans la Figure 2.

### Lieu de résidence de l'adhérent

L'utilisation des services de santé par des adhérents à la MUSA est plus importante chez ceux résidant dans les communes de Bagira (85%) et Kadutu (62%) tandis qu'elle est faible chez des adhérents résidents dans la commune d'Ibanda (37%). Le lieu de résidence peut en effet traduire un certain niveau socio-économique, notamment le degré de pauvreté. Les habitants de la commune de Bagira connaissent plus de pauvreté que ceux des autres communes [20] et l'association entre la pauvreté et la maladie est largement documentée [3, 4, 21, 22]. Cette raison pourrait justifier le nombre important des adhérents de Bagira qui ont utilisé les services de santé au cours de la période d'étude. Le lieu de résidence peut également être plus proche des formations sanitaires affiliées à la MUSA et offrant une meilleure qualité des soins, facilitant ainsi un accès aux services [23].

### Niveau d'instruction du responsable du ménage

Le niveau d'instruction constitue un des déterminants de la bonne utilisation des services de santé [24-26] qu'on soit un adhérant à la MUSA ou pas. Les résultats de la présente étude prouvent que les enfants des parents qui ont atteint au moins le secondaire comme niveau de scolarité ont plus utilisé les services de santé que ceux des parents ayant un niveau inférieur ou égale au primaire. Le niveau d'étude peut cependant être un facteur de confusion, corrélé avec le niveau de pauvreté en fonction du lieu de résidence.

### Expérience antérieure des soins dans la structure sanitaire partenaire de la MUSA

Dans notre étude, l'insatisfaction par rapport à la qualité des soins a découragé 85,9% des répondants à revenir aux soins dans la structure allouée par la MUSA. Une étude longitudinale dans une communauté rurale de la RD Congo avait montré que l'utilisation des services de santé avait diminué de près de 40% sur 5 ans (1987-1991) et que 18 à 32% de cette diminution s'expliquait par le coût [27]. En adhérent à la MUSA, la barrière financière d'accès aux soins est levée et devrait créer ainsi une meilleure utilisation des services comme cela a été démontré ailleurs [28], ce qui n'a pas été le cas dans notre étude. Ainsi, mettre en place une MUSA ne suffit pas pour garantir un accès aux soins de qualité aux adhérents, il faudrait tenir compte des besoins de la population et améliorer la performance des services de santé [29]. L'approvisionnement régulier en médicaments et l'amélioration de la qualité technique des services, la qualification technique du personnel et la qualité des infrastructures constituent également d'autres facteurs pouvant influencer les adhérents à utiliser les services de santé, de même que les qualités interpersonnelles du personnel soignant [27]. En effet, il a été montré que le comportement des agents de santé est déterminant pour fidéliser les utilisateurs des services de santé [30].

### Qualité des soins dans la structure partenaire des MUSA

Nos résultats montrent que 109 répondants sur 268, soit seulement 40% se sont dits insatisfaits. Parmi ceux qui se sont dits satisfaits, 91% d'entre eux n'ont plus utilisé ces services. L'insatisfaction ne permet pas de créer un climat de confiance avec les utilisateurs des services de santé concernés, de sorte qu'ils n'y retournent pas après une première utilisation [30]. Nos résultats ont montré que 65,3% des problèmes sanitaires ont été globalement pris en charge par les structures sanitaires partenaires. L'incapacité à prendre en charge certains problèmes des adhérents génère de l'insatisfaction. Cette incapacité des formations sanitaires partenaires est essentiellement due à la pénurie de certains médicaments spécifiques et au manque de certains équipements dont l'oxygénateur (raison évoquée par 22% des répondants). Face à cette situation, les patients n'hésitent pas à recourir à l'automédication (50% des cas), à des chambres de prière, au traitement traditionnel ou aux formations sanitaires non partenaires aux MUSA. Ce comportement corrobore le résultat d'une étude menée à Kinshasa [23].

### Capacité des ménages à payer le ticket modérateur

La présente étude démontre que l'utilisation de service de santé dépend de la capacité du ménage à payer le ticket modérateur (OR = 4,1(2,2-9,9)). Plusieurs études antérieures ont montré que l'abolition des frais des soins augmentait l'utilisation des services de santé [31, 32] même si elle a montré ses limites dans des pays à faible revenu, notamment la difficulté à recouvrir les coûts pour assurer la continuité du fonctionnement des services [32, 33]. La MUSA, par l'instauration du ticket modérateur, exclurait la frange la plus pauvre de la population, compromettant ainsi la couverture universelle en santé prônée par l'OMS [34-36]. La MUSA étant considérée dans l'imaginaire collectif de Bukavu comme destinée aux pauvres, ceux-ci y adhèrent et peinent ainsi à payer le ticket modérateur proposé. La MUSA devrait constituer un mécanisme d'une réelle solidarité de manière à repartir les charges couvertes par l'assurance en fonction des richesses des adhérents et permettre ainsi une couverture réellement universelle des soins. La solidarité ne fonctionne pas encore dans la ville de Bukavu [37], d'où la faible utilisation des services de santé par certains adhérents à la MUSA.

## Conclusion

La MUSA favorise l'accès aux soins de santé du fait qu'elle lève la barrière financière. La présente étude confirme qu'au-delà de la barrière financière, le développement d'une mutuelle de santé doit être accompagné d'une amélioration de la qualité des formations sanitaires partenaires, d'une meilleure maîtrise de l'ensemble des coûts pour la viabilité financière de la MUSA et de la lutte contre la sélection adverse en renforçant la sensibilisation à l'adhésion aux MUSA de tous les membres de famille dont ceux qui ne tombent pas souvent malade. Ce développement gagnerait à prendre en compte le lieu de résidence des adhérents, le niveau d'étude des chefs des ménages et la qualité des soins dans des structures partenaires pour satisfaire les besoins en soins des adhérents. Dans des contextes similaires à ceux de Bukavu [1, 16, 17], ces facteurs qui ressortent de l'étude en tant que principaux déterminant de l'utilisation des services de santé des adhérents à une MUSA ne sont pas souvent pris en compte lors de l'implantation des MUSA.

### Etat des connaissances actuelles sur le sujet

- Les déterminants d'adhésion à une mutuelle de santé dans des pays africains;
- Les mécanismes de fonctionnement des mutuelles de santé en Afrique centrale;
- Les taux de pénétration des mutuelles de santé dans plusieurs pays d'Afrique.

### Contribution de notre étude à la connaissance

- L'étude identifie les principaux déterminants de l'utilisation des mutuelles de santé dans une ville urbaine, en RD Congo;
- L'étude recommande de tenir compte de ces déterminants lors de l'implantation d'une mutuelle de santé;
- Au-delà de la barrière financière, l'étude atteste que le développement d'une mutuelle de santé doit être accompagné d'une amélioration de la qualité des formations sanitaires partenaires, d'une meilleure maîtrise de l'ensemble des coûts pour la viabilité financière de la MUSA et de la lutte contre la sélection adverse en renforçant la sensibilisation à l'adhésion aux MUSA de tous les membres de famille dont ceux qui ne tombent pas souvent malade.

## Conflits d'intérêts

Les auteurs ne déclarent aucun conflit d'intérêts.
